# High-dose vitamin D substitution in patients with COVID-19: study protocol for a randomized, double-blind, placebo-controlled, multi-center study—VitCov Trial

**DOI:** 10.1186/s13063-022-06016-2

**Published:** 2022-02-04

**Authors:** Fabienne Jaun, Maria Boesing, Giorgia Lüthi-Corridori, Kristin Abig, Anja Makhdoomi, Nando Bloch, Christina Lins, Andrea Raess, Victoria Grillmayr, Philippe Haas, Philipp Schuetz, Luca Gabutti, Jürgen Muser, Anne B. Leuppi-Taegtmeyer, Stéphanie Giezendanner, Michael Brändle, Jörg D. Leuppi

**Affiliations:** 1 University Clinic of Medicine, Cantonal Hospital Baselland, Rheinstrasse 26, CH-4410 Liestal, Switzerland; 2 Cantonal Hospital St. Gallen, Rohrschacherstrasse 95, CH-9001 St. Gallen, Switzerland; 3 Science Application Concepts GmbH, Luzernerstrasse 190, CH-6402, Merlischachen, Switzerland; 4 Cantonal Hospital Aarau, Tellstrasse 25, CH-5001 Aarau, Switzerland; 5 Regional Hospital Bellinzona, Via A. Gallino 12, CH-6500 Bellinzona, Switzerland; 6 Cantonal Hospital Baselland, Rheinstrasse 26, CH-4410 Liestal, Switzerland; 7 Centre for Primary Health Care, University of Basel, Rheinstrasse 26, CH-4410 Liestal, Switzerland

**Keywords:** Coronavirus, SARS-CoV-2, COVID-19, Vitamin D, Vitamin D deficiency

## Abstract

**Background:**

The coronavirus disease 19 (COVID-19) pandemic has caused millions of deaths, and new treatments are urgently needed. Factors associated with a worse COVID-19 prognosis include old age (> 65 years), ethnicity, male sex, obesity, and people with comorbidities. Furthermore, vitamin D deficiency was reported as a predictor of poor prognosis in patients with acute respiratory failure due to COVID-19. According to a recent clinical case series, vitamin D deficiency is a modifiable risk factor, which has the prospect of reducing hospital stay, intensive care, and fatal outcomes. Vitamin D has potent immunomodulatory properties, and its supplementation might improve important outcomes in critically ill and vitamin D-deficient COVID-19 patients. Despite the evidence that supports an association between vitamin D deficiency and COVID-19 severity, there is uncertainty about the direct link. Therefore, the aim of the trial is to assess if high-dose vitamin D supplementation has a therapeutic effect in vitamin D-deficient patients with COVID-19.

**Methods:**

As the trial design, a randomized, placebo-controlled, double-blind, multi-center approach was chosen to compare a high single dose of vitamin D (140,000 IU) followed by treatment as usual (TAU) (VitD + TAU) with treatment as usual only (placebo + TAU) in patients with COVID-19 and vitamin D deficiency.

**Discussion:**

Vitamin D substitution in patients with COVID-19 and vitamin D deficiency should be investigated for efficacy and safety. The study aim is to test the hypothesis that patients with vitamin D deficiency suffering from COVID-19 treated under standardized conditions in hospital will recover faster when additionally treated with high-dose vitamin D supplementation. Latest studies suggest that vitamin D supplementation in patients with COVID-19 is highly recommended to positively influence the course of the disease. With this randomized controlled trial, a contribution to new treatment guidelines shall be made.

**Trial registration:**

ClinicalTrials.gov NCT04525820 and SNCTP 2020-01401

## Administrative information

Note: the numbers in curly brackets in this protocol refer to SPIRIT checklist item numbers. The order of the items has been modified to group similar items (see http://www.equator-network.org/reporting-guidelines/spirit-2013-statement-defining-standard-protocol-items-for-clinical-trials/).

**Table:** 

Title {1}	High Dose Vitamin-D-substitution in patients with COVID-19: study protocol for a randomized, double blind, placebo controlled, multi-centre study- VitCov Trial
Trial registration {2a and 2b}.	Clinicaltrials.gov: NCT04525820 (from 25.August 2020) SNCTP: 2020-01401
Protocol version {3}	Version 3.1 26.07.2021
Funding {4}	The study received funding from SWICA AG, Foundation Bank Vontobel, SWF Foundation, Gebro Pharma AG and the by the internal funds of Professor Leuppi.
Author details {5a}	**Fabienne Jaun, BScN:** Co-Investigator, University Clinic of Medicine, Cantonal Hospital Baselland (corresponding author) **Maria Boesing: MD**, Study Physician University Clinic of Medicine, Cantonal Hospital Baselland and University of Basel **Giorgia Lüthi-Corridori, MSc**: Co-Investigator, University Clinic of Medicine, Cantonal Hospital Baselland and University of Basel **Kristin Abig:** Co-Investigator, University Clinic of Medicine, Cantonal Hospital Baselland **Maria Boesing: MD**, Study Physician University Clinic of Medicine, Cantonal Hospital Baselland and University of Basel **Anja Makhdoomi**: **MD,** University Clinic of Medicine, Cantonal Hospital Baselland and University of Basel **Nando Bloch: MD,** Study Phyisician, Cantonal Hospital St. Gallen **Christina Lins: MD,** Study Phyisician, Cantonal Hospital St. Gallen **Andrea Raess.** Local Study Coordinator, Cantonal Hospital St. Gallen **Victory Grillmayr,** Local Study Coordinator, Cantonal Hospital St. Gallen **Philippe Haas, MD, PhD,** Co-Investigator, Cantonal Hospital Baselland **Professor Philipp Schütz, MD**: Local Principal Investigator, Cantonal Hospital Aarau and University of Basel **Professor Michael Brändle, MD, MSc**: Local Principal Investigator, Cantonal Hospital St. Gallen **Professor Luca Gabutti, MD**: specialist advisor, Cantonal Hospital Bellinzona **Jürgen Muser, PhD**: specialist advisor, Cantonal Hospital Baselland **Prof. Anne B. Leuppi-Taegtmeyer, MD, PhD**: Clinical Pharmacologist & Toxicologist, Cantonal Hospital Baselland and University Hospital Basel **Stéphanie Giezendanner, PhD:** Trial Statistician, Centre for Primary Health Care, University of Basel **Professor Michael Brändle, MD, MSc**: Local Principal Investigator, Cantonal Hospital St. Gallen **Professor Jörg D. Leuppi, MD, PhD**: Sponsor, Principal Investigator. University Clinic of Medicine, Cantonal Hospital Baselland and University of Basel
Name and contact information for the trial sponsor {5b}	Prof. Jörg D. Leuppi, MD, PhD Clinical Professor of Internal Medicine, University of Basel Head of the University Clinic of Medicine cantonal hospital Baselland Rheinstrasse 26 CH-4410 Liestal Phone: + 41-61-925-21-80 E-Mail: joerg.leuppi@ksbl.ch
Role of sponsor {5c}	Professor Jörg D. Leuppi and his research group wrote the study protocol together with collaborating partners. The study will be conducted under the supervision of Prof. Leuppi. He and his research team are responsible for all submissions to obtain study approval from local authorities (ethical committee and Swissmedic). Prof. Leuppi is involved in every step of this study including data collection, interpretation of results and writing of scientific reports.

## Introduction

### Background and rationale {6a}

A new coronavirus (CoV) infection epidemic began in Wuhan, Hubei, China, in late 2019, originally called 2019-nCoV [1] and renamed COVID-19 by the World Health Organization in 2020. Previous CoV epidemics included severe acute respiratory syndrome (SARS)-CoV in 2003 [2] and Middle East respiratory syndrome (MERS)-CoV in 2012 [3]. The mortality rates were > 10% for SARS and > 35% for MERS [4]. So far, this pandemic is responsible for millions of deaths. The direct cause of death is generally due to ensuing severe atypical pneumonia, followed by acute respiratory distress syndrome (ARDS) [4, 5].

Risk factors for a poor outcome of SARS-CoV-2 infection have so far been identified to include older age (> 65 years) and comorbidities such as chronic respiratory conditions, hypertension or diabetes, obesity, current smoking status, male sex, and Black or Asian ethnic origin [6–9].

Observational studies reported independent associations between low serum concentrations of 25-hydroxyvitamin D (the major circulating vitamin D metabolite) and susceptibility to acute respiratory tract infection [10–12]. 25-Hydroxyvitamin D supports induction of antimicrobial peptides in response to both viral and bacterial stimuli [6–8, 13], suggesting a potential mechanism by which vitamin D-inducible protection against respiratory pathogens might be mediated [14]. Previous studies observed that vitamin D deficiency is prevalent among patients with ARDS and other chronic respiratory diseases and likely contributes to its pathogenesis through alveolar-capillary damage [15, 16]. It was further reported that vitamin D is a predictor of poor prognosis in patients with acute respiratory failure due to COVID-19 [17–19]. Therefore, leading experts in the field have called for well-powered randomized controlled trials of vitamin D supplementation as an add-on treatment for COVID-19 to test for causality [20].

A randomized placebo-controlled trial of high-dose vitamin D in critically ill adult patients with vitamin D deficiency found a significant in-hospital mortality reduction among patients with severe vitamin D deficiency compared to placebo [21]. In a further study of vitamin D status in patients with ARDS, Park and colleagues retrospectively examined data from 108 patients with ARDS for whom a vitamin D status was available; they observed that over 95% of these patients had vitamin D deficiency [22]. When examined according to quartile of serum 25-hydroxyvitamin D, a consistent inverse relationship between serum 25-hydroxyvitamin D and length of hospital and ICU stay among survivors was observed [22].

A treatment with vitamin D showed a significant reduction of inflammatory markers, a shorter time to recovery for patients with cough, gustatory sensory loss in patients with COVID-19, and vitamin D deficiency, without the occurrence of side effects [23, 24]. The anticipated risk of side effects due to a single high dose of vitamin D in this study is minimal, compared to the potential impact on preventing more extended hospital stays and worse outcomes in COVID-19. A single high dose of vitamin D in addition to smaller daily doses is secure and can be added to the current treatment guidelines for COVID-19 [23]. Vitamin D supplementation is a safe and cost-effective intervention, with multiple health benefits for patients with COVID-19 and vitamin D deficiency [25].

### Objectives {7}

The primary objective of this study is to investigate if a single high dose of vitamin D, in addition to treatment as usual (TAU), reduces the length of the hospital stay in patients with COVID-19 and vitamin D deficiency. Furthermore, we have defined the following parameters as secondary outcomes: the necessity of ICU treatment, overall mortality, percentage of patients with 25-hydroxyvitamin D > 50 nmol/L (> 20 ng/mL) at day 7, changes in serum calcium, phosphorus, 25-hydroxyvitamin D, parathyroid hormone (PTH), and the development of sepsis. We hypothesized that time to recovery is shorter in the single high-dose vitamin D group relative to the standard treatment group.

## Methods: participants, interventions, and outcomes

### Trial design {8}

We decided to conduct the study as a randomized, placebo-controlled, double-blind trial. This study compares a single high dose of vitamin D in addition to treatment as usual (VitD + TAU) to placebo and treatment as usual only (placebo + TAU). Ethically, it is not justifiable not to treat a known vitamin D deficiency. Therefore, we decided to compare the intervention (single high dose of vitamin D) with treatment as usual (smaller daily dose of vitamin D). The study is designed as a superiority study that assumes, testing the hypothesis, that a single high dose of vitamin D in addition to TAU leads to faster recovery than treatment as usual only.

### Study setting {9}

It is planned to conduct the study in four cantonal hospitals in Switzerland—two in north-western Switzerland, one in the canton of Tessin, and one in eastern Switzerland. All hospitals are category A clinics; two of them are academic. If necessary, other hospitals are asked to participate.

### Eligibility criteria {10}

Participants are eligible to participate in the study after giving written, informed consent when hospitalized on a general medicine ward due to ongoing, PCR-confirmed SARS-CoV-2 infection, aged ≥18 years with laboratory-confirmed vitamin D deficiency defined as a serum 25-hydroxyvitamin D concentration ≤ 50 nmol/L (≤20 ng/mL). Patients will be included independently of the severity of the disease.

The presence of any of the following conditions will lead to the exclusion of the participant: known hypersensitivity to one of the vitamin D products used in this study or to one of the adjuvants in the drug’s composition, active malignancy, hypercalcemia, granulomatous diseases such as sarcoidosis, history of renal stones within the past year, pregnancy/breastfeeding, previous enrolment into the current project, or another interventional trial.

### Who will take informed consent? {26a}

After a patient is identified as a potential subject, all inclusion criteria are fulfilled, and none of the exclusion criteria is present, the patient will be asked by a study physician if they want to participate in this study. The patient then needs to give his written informed consent before the randomization. The study information and consent form are provided to the patient by the physician, and both documents are revised and approved by the responsible local ethics committee.

### Additional consent provisions for collection and use of participant data and biological specimens {26b}

It is not planned to collect data or biological material from participants, which is not described or mentioned in the study protocol or patient information. If there are changes in the protocol, participating subjects are provided with a new informed consent form to decide if they want to participate in further investigations.

### Interventions

#### Explanation for the choice of comparators {6b}

This study will be conducted as a randomized, placebo-controlled, double-blind trial comparing high-dose vitamin D in addition to TAU with placebo + TAU. The recommended supplementation of vitamin D in adults is 800 IU vitamin D3/day [26, 27].

As there is no ethical justification for not supplementing vitamin D in patients with laboratory-confirmed vitamin D deficiency, we decided against a “placebo only” design. Instead, we will compare single high-dose vitamin D + TAU versus placebo + TAU.

#### Intervention description {11a}

##### Intervention group

Participants will receive a single oral dose of 140,000 IU of vitamin D3 as an oily solution and then continue with the standard oral dose of 800 IU vitamin D3 per day until discharge. The medication will be administered by instructed personnel—preferably in the morning along with the patients’ other prescribed medication. We did not find information about significant differences resulting from the timing of administration.

##### Control group

Participants will receive a single dose of placebo orally and then continue with the standard dose of 800 IU vitamin D3 per day. The procedure of administration is the same as in the intervention group. The placebo solution is similar to the product used in the intervention group (VITAMIN D3 Wild Öl 500 IU/drop) in terms of consistency and look. Neither patients nor physicians can detect a difference between the two products.

#### Criteria for discontinuing or modifying allocated interventions {11b}

Treating doctors can change the dose of vitamin D or stop the treatment if clinically indicated. In such cases, it must be reported to the lead center immediately. The patient will be considered as a dropout. The treating physician can re-evaluate the treatment throughout the trial and independently decide about additional treatment options. The patient must be informed about all treatment options and must be treated according to the most recent local or national COVID-19 guidelines.

#### Strategies to improve adherence to interventions {11c}

Patient adherence is not assessed since the participants are all hospitalized, and qualified personnel will administer medication. The nurses and doctors will ensure that the patient receives the study medication correctly. If there is any complication with the administration (e.g., vomiting after taking the study medication), it must be reported directly to the lead center. The local study team will collect the used medication bottles and, if needed, can be used for additional evaluation of adherence after the patient is discharged.

### Relevant concomitant care permitted or prohibited during the trial {11d}

All treatments or medication considered necessary by treating doctors are permitted, and their use will be recorded in the case report form. There is no restriction in using other treatments or interventions during this trial.

### Provisions for post-trial care {30}

Patients are carefully followed up until discharge from hospital care. Any after-care necessary due to COVID-19 is organized by the treating physicians and is not connected to this trial. This trial has a mandatory, trial-specific insurance during the whole study period.

### Outcomes {12}

As the primary outcome, we fixed the length of the hospital stay. Therefore, the measurement will be the overall duration of the hospitalization from enrolment until discharge from hospital care. As defined, to measure the secondary objectives, we will assess if there are any differences between the TAU + VitD and the TAU + placebo group in terms of the variables described in the table below.

**Table:** 

ICU stay	Yes/no
	If yes, length of the ICU stays (admission to discharge)
	Requirement for mechanical ventilation
Overall mortality	
Vitamin D serum concentration	% of patients with 25-(OH)D > 50 nmol/L at day 7
	25-(OH)D
Laboratory parameters	Calcium
	Phosphorus
	Parathyroid hormone (PTH)
Sepsis	% of patients who develop a sepsis
Complications	Complications during the hospitalization due to COVID-19
Vital signs	Blood pressure (BP)
	Heart rate (HR)
	Respiratory rate (RR)
	Blood oxygen saturation
	Need of oxygen and how much
Quality of life (SF-12 Questionnaire)	At baseline, 28 days, and 3 months after inclusion

### Participant timeline {13}

### Sample size {14}

To calculate the sample size, we looked at an interventional study of vitamin D, which examined the length of hospital stay as an outcome and what is known about the median length of hospital stay in patients hospitalized with COVID-19. A randomized, double-blind, placebo-controlled trial examined the effects of adjunctive vitamin D in adults hospitalized with community-acquired pneumonia (CAP) on time to recovery [28]. The primary outcome was the complete resolution of chest radiograph infiltrates at 6 weeks post-study treatment. Secondary outcomes included length of hospital stay, intensive care admission, and return to regular activity. At the same time, adjunctive vitamin D did not affect the primary outcome (*OR* 0.78, 95% *CI* 0.31 to 1.86, *p* = 0.548). There was evidence it increased the complete resolution of pneumonia in participants with baseline vitamin D levels < 25 nmol/L (*OR* 17.0, 95% *CI* 1.40–549.45, *p* = 0.043). Although not significant, the results indicated that patients in the intervention group had shorter length of hospital stay (mean = 3.8, *SD* = ± 3.1, *n* = 60) when compared with patients in the placebo group (mean = 4.8, *SD* = ± 6.5, *n* = 57), which indicates a small effect size (Cohen’s *d*) of 0.1980. The study indicated a 20% reduction in length of hospital stay.

Considering the above results in the light of COVID-19, more extended hospital stays in COVID-19 patients are expected. In smaller observational studies describing the clinical course of hospitalized COVID-19 patients in China, a median hospital stay of 12 days (*IQR* 9–15) (*n* = 137) [1] or of 10 days (*IQR*, 7.0–14.0) among those discharged alive (*n* = 47) [29] were observed. Guan et al. presented the clinical characteristics of 1099 patients with laboratory-confirmed COVID-19, and the median length of hospital stay was reported to be 12 days (*IQR* = 10–14) [30], which we will take as a basis for our sample size calculation.

Sample size calculation was performed using the function “power.t.test” from the R package “stats” with the following parameters [31]: alpha = 0.05, power = 0.80, sigma = 2.96, delta (difference in the primary outcome between intervention and control group) = 2, and alternative hypothesis = two-sided, which resulted in a minimum of 35 patients per group, therefore a total of 70 patients. With a conservatively estimated 10% dropout rate, including the loss to follow-up, and a block size randomization of four, we decided to include 80 patients.

### Recruitment {15}

After a patient is identified as a potential participant, all inclusion criteria are fulfilled, and none of the exclusion criteria is present, the patient will be asked by their treating physician if they want to participate in this study.

Only patients on general wards will be asked. Patients who are already being treated in the intensive care unit or taking part in another interventional trial will not be considered as potential participants. Neither patients nor doctors will receive financial reimbursement. The study budget will cover expenses for the study medication and diagnostics performed only during this study. The recruitment will take place in the described way in all four centers (cantonal hospital of Baselland in Liestal and Bruderholz, the cantonal hospital of Aarau, the cantonal hospital of St. Gallen, and the regional hospital Lugano). A collaboration with other hospitals in Switzerland will be considered if recruitment is slow.

### Assignment of interventions: allocation

#### Sequence generation {16a}

Participants will be randomly assigned to either the intervention or the control group in a 1:1 ratio. A statistician not involved in the data analysis generates a randomization list with study group allocation using R [31]. Randomization is carried out stratified by center using block randomization with a block size of four to account for differences in standard of care. The randomization master list will be handed over to the university hospital pharmacy in Basel, where the study drug will be labeled and packaged accordingly. In case of an adverse event (AE) or serious adverse event (SAE) that requires an emergency code break, the hospital pharmacy can decode the randomization immediately.

#### Concealment mechanism {16b}

The hospital pharmacy of the university hospital of Basel will pack and label the study medication according to the randomization master list. They will provide the study centers with sealed, identically looking medication packages for each patient that contains either the high-dose vitamin D and the standard medication or the placebo and the standard medication. Participating centers will receive a certain number of blocks of the study drug, which must be used strictly in the ascending order indicated by the code.

#### Implementation {16c}

The treating physician will make the enrolment. After a patient has given his/her consent, the physician will take one of the prepared medication packages with the medication. As described above, the assignment to either group will be made randomly.

### Assignment of interventions: blinding

#### Who will be blinded {17a}

As the hospital pharmacy provides the medication in sealed, identically looking medication packages, neither the treating physician nor the patient or other care providers knows if the patient is in the intervention or placebo group. The statistician will conduct the analysis blinded.

#### Procedure for unblinding if needed {17b}

In case of an event or medical emergency that makes unblinding necessary, the hospital pharmacy can perform a patient-specific unblinding at any time during the study. In such a case, treating doctors can call the pharmacist who has access to the concealed list. Based on the study design and the intervention in this trial, it is assumed that no emergency code break will be necessary. In case unblinding becomes necessary, collected patient data will be used for the primary analysis in an intention-to-treat approach, but the analyzing statistician will remain blinded.

### Data collection and management

#### Plans for assessment and collection of outcomes {18a}

All outcomes are assessed by treating physicians or nurses until discharge or fatality. The CRF is provided either electronically (Web-based) or in paper form, in which case it will be sent by mail or fax to the coordinating study center, where data will be entered into the Web-based database.

The primary outcome is the length of hospital stay. The exact length will be calculated automatically by the electronic database into which only the date of the admission, the date of inclusion in the study, and the date of discharge must be entered. With this method, copying or calculation errors can be minimized. Patients are screened daily and therefore we will have only a small delay of approximately 1 day from hospital admission until inclusion to the study.

Secondary outcomes are the following:

**Table:** 

ICU stay	Yes/no
	If yes, length of the ICU stays (admission to discharge)
	Requirement for mechanical ventilation
Overall mortality	
Vitamin D serum concentration	% of patients with 25-(OH)D > 50 nmol/L at day 7
	25-(OH)D
Laboratory parameters	Calcium
	Phosphorus
	Parathyroid hormone (PTH)
Sepsis	% of patients who develop a sepsis
Complications	Complications during the hospitalization due to COVID-19
Vital signs	BP
	HR
	RR
	Blood oxygen saturation
	Need of oxygen and how much
Quality of life (SF-12 Questionnaire)	At baseline, 28 days, and 3 months after inclusion

The treating physicians will provide the research team with a copy of all laboratory results (anonymized, with participant identification number) as source data. This procedure was chosen to avoid transcription errors in the paper-based CRF or the electronic CRF.

#### Plans to promote participant retention and complete follow-up {18b}

Participants can withdraw from the study at any time without stating reasons. Collected data and samples are evaluated in encrypted form until the withdrawal of consent. The data from patients who have withdrawn consent will be used to analyze in a modified intention-to-treat approach, explained in detail in section statistical methods for primary and secondary outcomes.

### Data management {19}

Data acquisition will be performed by participating centers, entering data directly through a Web-based electronic case report form (eCRF) or by sending a paper version of the CRF to the research team at the main study center (Kantonsspital Baselland, Liestal), where data entry is performed by one of the team members. Data from paper CRFs entered at the study center will be double-checked for data verification purposes. Data about adverse events will be reported by participating centers to the research team at the lead center. The lead center will inform the relevant authorities (ethics committee and the Swissmedic).

Data entered into the electronic CRF or paper version of the CRF will be treated as source data.

All study data will be archived at the lead study center in Liestal for a minimum of 10 years after study termination or premature termination of the clinical trial.

The data will be collected in a Web-based electronic data capture (EDC) system named secuTrial®. The system is accessible via a standard browser and password protection ensures that only authorized personnel can enter the system to view, add, or edit data according to their permissions. Back up of secuTrial® study data is performed according to the IT department of the university hospital Basel.

The EDC will be locked after the data is monitored and all raised queries have been resolved. Data is exported and transferred to the investigator by the CTU according to internal defined processes. Data will be archived for a minimum of 10 years by the sponsor-investigator. Data is validated for completeness and discrepancies automatically. An audit trail maintains a record of initial entries and changes (reasons for changes, time and date of changes, user identification of entries and changes). The data entered into the eCRF will be reviewed by the responsible centers and an independent monitor will raise queries using the query management system implemented. The participating center must respond to the query and confirm or correct the corresponding data. After that, the monitor can close the query.

### Confidentiality {27}

All collected data will be treated as confidential. The participating center and data will be anonymized; all data will be stored and analyzed in an anonymized way. The results of this study will be published in an anonymized fashion. Direct access to source documents will be permitted for purposes of monitoring, audits, and inspections. The study protocol and dataset shall be accessible to any regulatory authority after publication for at least 10 years.

### Plans for collection, laboratory evaluation, and storage of biological specimens for genetic or molecular analysis in this trial/future use {33}

We have no plans for collecting, laboratory evaluation, and storage of any biological specimens for genetic or molecular analyses in this trial or further use. This trial only uses blood samples taken routinely during the hospitalization of the patients, and no other samples of any other biological material are planned.

### Statistical methods

#### Statistical methods for primary and secondary outcomes {20a}

A statistical analysis plan documented detailed methodology for summaries and statistical analyses of the data collected in this trial. The statistical analysis plan is finalized analysis [31]. We will summarize our results using descriptive statistics.

The primary analysis comparing the mean length of hospital stay between the two groups will be performed on the dataset of all randomized patients for whom the outcome measure is available. A two-sided independent Student’s *t*-test will be used following log-normal transformation as Feng et al. [32] suggested. The time to discharge will be assessed up to 3 months, with failure to reach discharge or die before that day considered as right censored at 3 months. Patients who are admitted to ICU or change wards within the hospital can continue the study and their data can be analyzed as planned, since the intervention can be continued, and daily visit data are available within the electronic patient documentation system. For patients who are transferred to another hospital, the primary endpoint will be taken from the respective discharge letter, which is by standard sent from the following hospital to the referring hospital. If this data is not available, the patient will be considered as right censored at the date of transfer.

In the secondary outcome analyses, mean levels of calcium, phosphorus, 25-hydroxyvitamin D, 1,25-dihydroxy vitamin D, and PTH will be compared using two-sided independent Student’s *t*-tests. To compare the proportion of patients who died, were admitted to ICU, developed sepsis, and as the proportion of patients with 25-hydroxyvitamin D ≥ 30 ng/mL at discharge between the two groups, either the χ ^2^ test or the Fisher exact probability test will be used. The Fisher exact probability test is an excellent non-parametric technique for comparing proportions when the two independent samples are small.

#### Interim analyses {21b}

Due to the relatively small sample size and short study duration, we will not identify stopping criteria nor perform interim analyses.

#### Methods for additional analyses (e.g., subgroup analyses) {20b}

To evaluate the safety of short-term vitamin D administration in the above-described population, linear/logistic mixed-effect models will be used to estimate treatment effects on safety parameters such as adverse events/laboratory parameters (e.g., serum calcium or PTH) and vital signs. If data permits, subgroup analyses will be performed, such as analyses stratified by gender or baseline vitamin D levels.

#### Methods in analysis to handle protocol non-adherence and any statistical methods to handle missing data {20c}

Careful trial planning and execution will minimize the occurrence of missing data as far as possible. In cases where data is missing, treating physicians will be contacted to complete the data from patients’ records. If none of the valid reasons to ignore missing data as cited in Jakobsen’s practical guide [33] is fulfilled, multiple imputation by chained equations will be performed using the R package “mice” [34]. A sensitivity analysis will be performed on the dataset of patients treated without major protocol deviations, such as delay or noncompliance with the medication plan. Any changes or deviations from the original statistical plan will be reported to the ethics committee.

#### Plans to give access to the full protocol, participant-level data, and statistical code {31c}

We plan to publish this protocol, meaning it will be accessible to the public. The complete statistical methods and results will also be published as soon as data collection and analysis are completed. Upon request, competent authorities (CA) will have full access to anonymized patient data and complete statistical code.

### Oversight and monitoring

#### Composition of the coordinating center and trial steering committee {5d}

Professor Leuppi at the medical university clinic of the cantonal hospital Baselland leads this study, which is the coordinating center. The multidisciplinary research team includes medical doctors, a clinical pharmacologist, study nurses, statisticians, and public health scientists. Furthermore, the study team is supported by the clinical trial unit from the clinical research department at the university hospital of Basel. A trial steering committee is not planned.

#### Composition of the data monitoring committee, its role, and reporting structure {21a}

All study data and documents shall be accessible to monitors, and questions will be answered during monitoring. The sponsor will perform the site initiation visits and the close-out visit. Scheduled monitoring time points are after the inclusion of the first ten patients and after the last visit of the last patient.

#### Adverse event reporting and harms {22}

##### Reporting of SAEs

An adverse event (AE) is defined as any untoward medical occurrence in a patient, or a clinical investigation participant administered a pharmaceutical product, which does not necessarily have a causal relationship with the study procedure. An AE can be any unfavorable and unintended sign (including an abnormal laboratory finding), symptom, or disease, whether or not related to the medicinal (investigational) product. A serious adverse event (SAE) is classified as any untoward medical occurrence that results in death, is life-threatening, requires in-patient hospitalization or prolongs existing hospitalization, results in persistent or significant disability/incapacity, or is a congenital anomaly/congenital disability.

In addition, critical medical events that may not be immediately life-threatening, result in death, or require hospitalization but may jeopardize the patient or require intervention to prevent one of the other outcomes listed above should also usually be considered serious.

All SAEs must be reported immediately and within a maximum of 24 h to the sponsor-investigator of the study. The sponsor-investigator will re-evaluate the SAE and return the form to the site. SAEs resulting in death are reported to the ethics committee via BASEC within 7 days.

All involved ethics committees receive SAEs resulting in death in Switzerland via sponsor-investigator via BASEC within 7 days.

##### Reporting of suspected unexpected serious adverse reactions (SUSARs)

A SUSAR needs to be reported to the ethics committee (local event via the local investigator) via BASEC and to Swissmedic for category B and C studies (via sponsor-investigator) within 7 days if the event is fatal, or within 15 days (all other events).

The sponsor-investigator must inform all investigators participating in the clinical study of the occurrence of a SUSAR. According to the exact timelines, all in the trial involved ethics committees will be informed about SUSARs in Switzerland via the sponsor-investigator via BASEC.

##### Reporting of safety signals

All suspected new risks and relevant new aspects of known adverse reactions that require safety-related measures, i.e., so-called safety signals, must be reported to the sponsor-investigator within 24 h. The sponsor-investigator must report the safety signals within 7 days to the ethics committee (local event via the local investigator) via BASEC and to Swissmedic in case of a category B or C study.

The sponsor-investigator must immediately inform all participating investigators about all safety signals. The other ethics committees involved in the trial will be informed about safety signals in Switzerland via the sponsor-investigator.

##### Reporting and handling of pregnancies

Pregnant participants must immediately be withdrawn from the clinical study. Any pregnancy occurring during the treatment phase of the study and within 30 days after discontinuation of study medication will be reported to the sponsor-investigator within 24 h. The administration of vitamin D3 is not contraindicated in pregnancy, but hypercalcemia caused by long-term overdose can lead to various negative outcomes for the child. Furthermore, since the evidence for the effect of high-dose vitamin D in patients with COVID-19 remains uncertain, it was decided to exclude pregnant women. Pregnant women with a known vitamin D deficiency should be supplemented according to prescribing information or according to the recommendations of the treating gynecologist.

##### Periodic reporting of safety

An annual safety report (ASR) is submitted once a year to the local ethics committee by the local investigator and to Swissmedic in case of a category B study, as this study is categorized, via the sponsor-investigator.

#### Frequency and plans for auditing trial conduct {23}

Independently from investigators, regulatory authorities can audit this trial. Study documentation and data are accessible to auditors/inspectors, and questions are answered during inspections. All involved parties must keep the participant data strictly confidential.

#### Plans for communicating important protocol amendments to relevant parties (e.g., trial participants, ethical committees) {25}

Substantial amendments are only implemented after approval of the local ethics committee (LEC) and Swissmedic, respectively.

Under emergency circumstances, deviations from the protocol to protect human subjects’ rights, safety, and well-being may proceed without prior approval of the sponsor and the LEC/CA. Such deviations shall be documented and reported to the sponsor and the LEC/CA as soon as possible. All non-substantial amendments are communicated to the CA as soon as possible.

#### Dissemination plans {31a}

We aim to publish the results of this study in a peer-reviewed journal. There is no intention to use professional writers. This trial is registered at www.clinicaltrials.gov and kofam.ch and is therefore accessible to the public.

## Discussion

The primary aim of this study is to determine whether a high dose of vitamin D improves the course of COVID-19 or not. Two issues might affect the ability to recruit patients for this trial: first levels of vitamin D are the highest by the end of summer [35], which could lead to problems in recruiting. After the full ethics approval and the approval from Swissmedic, the recruitment period is expected to start at end of 2020 or early 2021. Shorter days and less exposure to sunlight most certainly lead to a decreasing serum concentration of vitamin D. Therefore, we anticipate that we will not have recruiting problems due to high vitamin D levels.

Secondly, travel regulation and other policies can change day by day and are carefully monitored by the Swiss government, which leads to a more unpredictable situation for this and other studies investigating COVID-19.

While the number of patients hospitalized with COVID-19 declined between April and August, numbers have started to rise again. Latest studies have shown a link between international and domestic air travel and the number of COVID-19 cases, so unless air travel is restricted again, we can expect an increasing number of cases [36, 37]. The same can be applied for lockdown policies and other public health measures, leading to decreasing cases [38, 39] and subsequent rebounds after gradually releasing the measures.

There are minimal risks in the participation in this study. Almost no side effects are reported when using a high vitamin D dosage, but it has a potential short and may also long-term benefit [23, 24, 40]. Some recent findings indicate an association between vitamin D deficiency and disease severity [23, 41, 42]. However, there is a lack of consensus on the dosage of vitamin D supplementation [40]. With this trial, we hope to make a valuable contribution in clarifying the role of vitamin D in COVID-19 and its effect on short-term outcomes. The crucial point is that vitamin D supplementation is a simple, safe, and inexpensive possibility to correct vitamin D deficiency, with potential benefits to patients with COVID-19 [43]. On a larger scale, the benefit that effective treatment of vitamin D deficiency in patients with COVID-19 could have on the overall costs of the health care system must also be considered. The authors hope to make a valuable contribution to future treatment guidelines for patients with COVID-19.

## Trial status

This study was submitted to the local ethics committee the first time at the end of May 2020. After the feedback, we revised the study protocol to change the design from an open label to randomized controlled double-blind. Other changes were made in the protocol (version 3.1., 26.07.2021), which was approved by the regional ethics committee without further obligations in August 2021. This trial was also submitted to Swissmedic and got the approval in October 2020. We submitted this protocol to *Trials* first in November 2020, and revisions came back in September 2021. Neither the submitting authors nor the Editor of the Journal can explain what happened in the meantime. We could include the last patient for this trial in mid of September 2021. The follow-up (28 days and 3 months) quality of life will be terminated by mid-November 2021.

## Abbreviations

AE: Adverse event
ARDS: Acute respiratory distress syndrome
ASR: Annual safety report
BASEC: Business Administration System for Ethical Committees (https://submissions.swissethics.ch/en/)
BP: Blood pressure
CA: Competent authority (e.g., Swissmedic)
CRF: Case report form
CoV: Coronavirus
eCRF: Electronic case report form
CTU: Clinical trial unit
EKNZ: Ethical committee of north-western and central Switzerland
EKOS: Ethical committee of eastern Switzerland
EOC: Ente Ospidaliero Cantonale (Regional Hospital Ticino)
FAS: Full analysis set
HR: Heart rate
KSA: Kantonsspital Aarau
KSBL: Kantonsspital Baselland
KSSG: Kantonsspital St. Gallen
LEC: Local ethics committee
MERS: Middle East respiratory syndrome
PI: Principal investigator
PP: Per protocol
PTH: Parathyroid hormone
RR: Respiratory rate
SAE: Serious adverse event
SARS: Severe acute respiratory syndrome
SUSAR: Suspected unexpected serious adverse reaction
TAU: Treatment as usual
TI: Ticino
